# Direction-of-Arrival Estimation for Circulating Space-Time Coding Arrays: From Beamspace MUSIC to Spatial Smoothing in the Transform Domain

**DOI:** 10.3390/s18113689

**Published:** 2018-10-30

**Authors:** Huake Wang, Guisheng Liao, Jingwei Xu, Shengqi Zhu, Cao Zeng

**Affiliations:** National Laboratory of Radar Signal Processing, Xidian University, Xi’an 710071, China; gsliao@xidian.edu.cn (G.L.); xujingwei1987@163.com (J.X.); zhushengqi8@163.com (S.Z.); czeng@mail.xidian.edu.cn (C.Z.)

**Keywords:** circulating space-time coding array, space-time matched filter, transmit beamspace, Cramér-Rao lower bound, DOA estimation

## Abstract

As a special type of coherent collocated Multiple-Input Multiple-Output (MIMO) radar, a circulating space-time coding array (CSTCA) transmits an identical waveform with a tiny time shift. It provides a simple way to achieve a full angular coverage with a stable gain and a low sidelobe level (SLL) in the range domain. In this paper, we address the problem of direction-of-arrival (DOA) estimation in CSTCA. Firstly, we design a novel two-dimensional space-time matched filter on receiver. It jointly performs equivalent transmit beamforming in the angle domain and waveform matching in the fast time domain. Multi-beams can be formed to acquire controllable transmit freedoms. Then, we propose a beamspace multiple signal classification (MUSIC) algorithm applicable in case of small training samples. Next, since targets at the same range cell show characteristics of coherence, we devise a transformation matrix to restore the rotational invariance property (RIP) of the receive array. Afterwards, we perform spatial smoothing in element domain based on the transformation. In addition, the closed-form expression of Cramer-Rao lower bound (CRLB) for angle estimation is derived. Theoretical performance analysis and numerical simulations are presented to demonstrate the effectiveness of proposed approaches.

## 1. Introduction

Circulating space-time coding array (CSTCA) as a simple transmission diversity technique has drawn tremendous attention from researchers recently [1,2,3,4,5,6]. Unlike traditional colocated MIMO radar [7,8,9,10], CSTCA transmits a single waveform with a tiny time shift across array elements to acquire full spatial illumination.

Space-time coding is based on the colored space-time waveform transmission principle. It allows transmitting different waveforms in different directions with a wide angular coverage [11,12,13,14,15]. The performance of four typical space-time coding waveforms for active antenna systems was assessed by ambiguity functions in [3]. The impact of mutual coupling on MIMO radar with space-time coding was investigated in [4], and a calibration procedure for the transmit beamforming was presented. Particularly, the concept of circulating space-time coding was proposed in [1]. As a special type of coherent colocated MIMO radar [16], CSTCA is simple to be implemented in engineering [17,18]. In [17], a low-cost digital beamforming technique was introduced according to circulating signal principle. In [18], the element embedded pattern error and antenna coupling were directly calibrated in operating mode by exploiting circulating space-time coding. In [19], an extended circulating space-time code with constant modulus was suggested for transmit beampattern synthesis. The joint slow-time coding with circulating codes was derived to restore the range resolution and suppress interferences in [20].

Direction-of-arrival (DOA) estimation of multiple targets is one of the most important radar applications [21,22,23]. Methods in the context of colocated MIMO radar have been developed extensively. The most popular approaches are multiple signal classification (MUSIC) and estimation of signal parameters via rotational invariance techniques (ESPRIT) with high resolution capabilities [24,25,26,27]. However, MIMO radar demands to transmit orthogonal waveforms ensuring independence between sensor channels. The completely orthogonal waveforms are hardly available for multi-channel transmissions resulting in gain fluctuations in different directions. Besides, the correlations between distinct waveforms usually lead to a high sidelobe level in range domain, which has an adverse impact on weak target detection. Additionally, the constraints of constant modulus cannot be satisfied without performance loss in such cases [28,29].

The transmit beamspace design for colocated MIMO radar has been widely explored, which is generally formulated as an optimization problem with a tedious solving process [30]. Compared with current beamspace direction finding techniques [30,31,32], the proposed MUSIC algorithm can circumvent requirements on beamspace design. Besides, DOAs can be estimated properly in case of small samples with a low sidelobe level (SLL) in range domain. Therefore, CSTCA scheme is an advisable choice in parameter estimation, which has not been suggested in the literature.

The Cramér-Rao lower bound (CRLB) as a benchmark for unbiased estimators is of pivotal importance for target parameter estimation [33,34,35]. In [36,37], the CRLB of colocated MIMO radar was derived. However, the structure of the likelihood function is changed due to the intrinsic property of coupling between pulse compression and beamforming in CSTCA. Thus, the CRLB in CSTCA is different from that in MIMO radar and deserving of further analysis.

Unlike existing DOA estimation studies that mainly concentrate on the characteristics of the covariance matrix in receive beamspace [38,39,40,41,42], we focus on the transmit beamspace to estimate DOAs in CSTCA. Our main contributions include: (1) We design a novel two dimensional space-time matched filter incorporating pulse compression with beamforming. It can form a cluster of equivalent transmit multi-beams to obtain degree-of-freedoms (DOFs) in transmit domain. (2) The beamspace MUSIC method in CSTCA is proposed. We devise the searching steering vector in beamspace and derive the spatial spectrum. Moreover, since multi-targets in the same range cell appears characteristics of coherence, we design a transformation matrix which can turn data from beamspace into element space. The desired property of rotational invariance property (RIP) at the receive array can be guaranteed through transformation. Eventually, we estimate DOAs by spatial smoothing. (3) The closed-form expressions of Cramer-Rao lower bound for angle estimation is derived for the first time.

The remainder of this paper is organized as follows: the signal model of circulating space-time coding array is formulated in Section 2. In Section 3, we present the scheme of bi-dimensional space-time matched filtering. Additionally, the beamspace MUSIC method and the spatial smoothing method in transform domain are proposed in application of different scenarios. Experimental results demonstrating the effectiveness of the proposed algorithms are given in Section 4, followed by conclusions drawn in Section 5.

## 2. Signal Model of Circulating Space-Time Coding Array

Consider a uniform linear *M* element CSTCA with half-wavelength spacing *d*. An identical linear frequency modulation (LFM) waveform circulates through every channel. The tiny time shift Δ*t* is employed across array elements. The transmitted signal of the *m*th element can be expressed as:

(1) $$s_{m}(t)=e^{j2\pi f_{0}t}s(t-(m-1)\cdot \Delta t)$$

where *f*_0_ is the carrier frequency. The time shift is inversely proportional to signal bandwidth *B*. The transmitted waveform *s*(*t*) can be written as:

(2) $$s(t)=\mathrm{rect}\left(\frac{t}{T_{p}}\right)e^{j\pi \mu t^{2}}$$

where $\mathrm{rect}(\frac{t}{T_{p}})=\{\begin{array}{ccc} 1, & & 0\leq t\leq T_{p}\\ 0, & & \mathrm{else} \end{array}$ is the rectangular envelope, and *μ* = *B*/*T_p_* is the chirp rate with *T_p_* being the pulse duration. Furthermore, the time-variant waveform can be combined with the time-invariant spatial phase codes in space-time coding to improve the range resolution [2]. This kind of transmit diversity technique imposes an additional phase shift on the emitted signal. Meanwhile, the waveform itself is unchangeable. Herein, we associate LFM waveform as the time domain signal with Barker code ***c*** = [*c*_1_, *c*_2_, …, *c_M_*] as the spatial domain signal. Thus, the *m*-th hybrid signal can be represented by:

(3) $$s_{m}^{\prime }(t)=c_{m}s_{m}(t)$$

**Figure 1:**
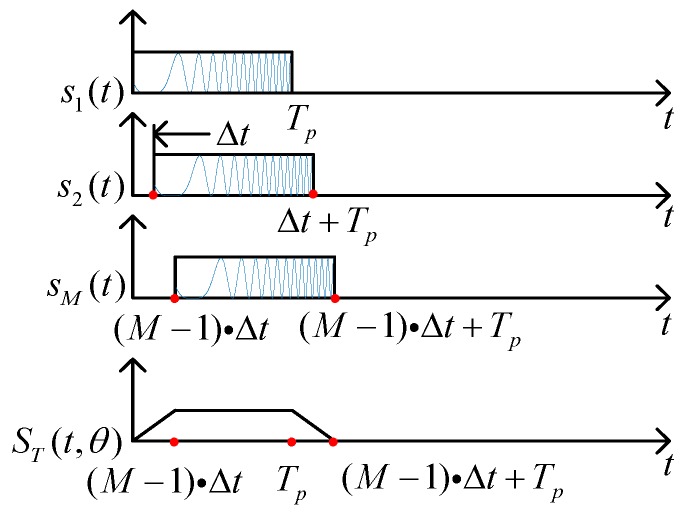
Sketch of circulating space-time coding LFM signal.

**Figure 2:**
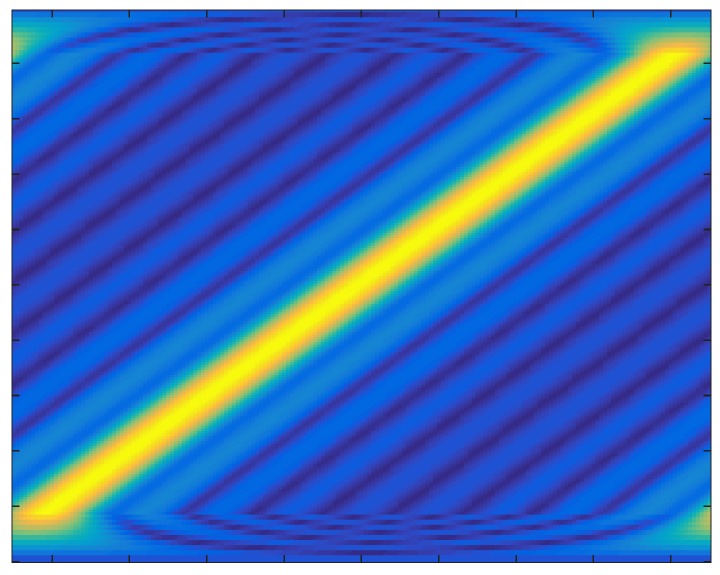
The transmit beampattern at *R* = 3 km in CSTCA.

The overall transmitted signal at angle *θ* in range *R* can be modelled as:

(4) $$S_{T}(t-\tau ,\theta )=e^{j2\pi f_{0}\left(t-\tau \right)}\sum_{m=1}^{M}e^{j2\pi \frac{d}{\lambda }(m-1)\sin\theta }\cdot s(t-(m-1)\cdot \Delta t-\tau )$$

where *τ* = *R*/*c* is the time delay. As illustrated in Figure 1, the transmitted signal is highly overlapped along the time axis. The envelope of overall signal is similar to the trapezoid with time duration *T_p_* in −3 dB level due to the transient effect. Besides, the pulse duration of overall signal is expanded into *T_p_* + (*M* − 1)Δ*t* leading to an edge effect [6]. Note that the transmitted signal of every element can’t be summed in any range cell, since Δ*t* = 1/*B* exactly corresponds to the size of a range resolution cell, that is, *c*/2*B.* Thus, CSTCA is capable of full spatial coverage with a range-angle- dependent transmit beampattern as shown in Figure 2. It is shown that the mainlobe points to different directions at distinct time instants during the pulse duration.

Without loss of generality, suppose *Q* far-field point targets at angles *θ*_1_, …, *θ_Q_* at ranges *R*_1_, …, *R_Q_*. After backscattering, the received signal at the *n*th element can be concisely written as

(5) $$y_{n}(t)=\sum_{q=1}^{Q}\zeta_{q}e^{j2\pi f_{0}\left(t-\tau_{q}\right)}e^{j2\pi \frac{d}{\lambda }(n-1)\sin\theta_{q}}\sum_{m=1}^{M}e^{j2\pi \frac{d}{\lambda }(m-1)\sin\theta_{q}}s(t-(m-1)\cdot \Delta t-\tau_{m,n,q})+n(t)$$

where *ξ_q_* denotes the complex scattering coefficient of the *q*th target, $\tau_{m,n,q}=\tau_{q}-d\left(m-1\right)\sin\theta_{q}/c-d\left(n-1\right)\sin\theta_{q}/c$ is the round-trip time delay with $\tau_{q}=2R_{q}/c$, *n*(*t*) is the additive Gaussian white noise with zero-mean. Under the assumption of a narrowband sounding signal, we have $s(t-\left(m-1\right)\Delta t-\tau_{m,n,q})\approx s(t-\left(m-1\right)\Delta t-\tau_{q})$. Therefore, (5) can be rewritten as

(6) $$y_{n}(t)\approx \sum_{q=1}^{Q}\zeta_{q}e^{j2\pi f_{0}\left(t-\tau_{q}\right)}e^{j2\pi \frac{d}{\lambda }(n-1)\sin\theta_{q}}\sum_{m=1}^{M}e^{j2\pi \frac{d}{\lambda }(m-1)\sin\theta_{q}}s(t-(m-1)\cdot \Delta t-\tau_{q})+n(t)$$

After down-conversion, the received signal from *N* receive channels can be concisely formulated as

(7) $$Y(t,\theta )=A_{R}\left(\theta \right)\left[w_{1}{\left(A_{T}\left(\theta \right)⊙S\left(t\right)\right)}^{T}w_{2}\right]+n(t)$$

where $A_{R}\left(\theta \right)=\left[a_{R}\left(\theta_{1}\right),a_{R}\left(\theta_{2}\right),\ldots ,a_{R}\left(\theta_{Q}\right)\right]$ is the *N* × *Q* receive array manifold with *N* × 1 receive steering vector $a_{R}\left(\theta_{q}\right)=\left[1,\exp\left\{j2\pi d\sin\theta_{q}/\lambda_{0}\right\},\cdots ,\exp\left\{j2\pi d(N-1)\sin\theta_{q}/\lambda_{0}\right\}\right]$, $A_{T}\left(\theta \right)=\left[a_{T}\left(\theta_{1}\right),a_{T}\left(\theta_{2}\right),\ldots ,a_{T}\left(\theta_{Q}\right)\right]$ is the *M* × *Q* transmit array manifold with *M* × 1 transmit steering vector $a_{T}\left(\theta_{q}\right)={\left[1,\exp\left\{j2\pi d\sin\theta_{q}/\lambda_{0}\right\},\cdots ,\exp\left\{j2\pi d(M-1)\sin\theta_{q}/\lambda_{0}\right\}\right]}^{T}$, $S\left(t\right)=\left[{\tilde{s}}_{1}\left(t\right),{\tilde{s}}_{2}\left(t\right),\cdots ,{\tilde{s}}_{Q}\left(t\right)\right]\in {\mathbb{C}}^{M\times Q}$ is the transmit waveform matrix with *M* × 1 waveform vector ${\tilde{s}}_{q}\left(t\right)={\left[s\left(t-\tau_{q}\right),s\left(t-\Delta t-\tau_{q}\right),\cdots ,s\left(t-(M-1)\Delta t-\tau_{q}\right)\right]}^{T}$, ***w***_1_ = diag(*ξ*_1_$e^{-j2\pi f_{0}\tau_{1}}$, …, *ξ_Q_*$e^{-j2\pi f_{0}\tau_{Q}}$) denotes a diagonal matrix with *q*th diagonal elements *ξ_q_*$e^{j2\pi f_{0}\tau_{q}}$, ***w***_2_ = [1, 1, …, 1]_*M*×1_, ***n***(*t*) is the *N* × 1 Gaussian white noise. ⊙ stands for the Hadamard product, (·)*^T^* denotes the transposition operation.

## 3. DOA Estimation for Circulating Space-Time Coding Array

### 3.1. Two-Dimensional Space-Time Matched Filtering Design

Based on the time-angle-dependent transmit beampattern in CSTCA, the conventional matched filter in fast time domain is no longer applicable. In this section, we devise a two-dimensional space-time matched filter to perform transmit beamforming and pulse compression simultaneously. The matched function can be formulated as:

(8) $$h(t,\theta_{i})=\sum_{n=1}^{M}e^{j2\pi \frac{d}{\lambda }(n-1)\sin\theta_{i}}s(t-(n-1)\cdot \Delta t)$$

where *θ_i_* is the matched angle, namely, the beamforming direction. It satisfies the following condition:

(9) $$\sin\theta_{i}=-1+2\left(i-1\right)/M,\;i=1,\ldots ,M$$

Under the constraint of (9), the steering vector can be turned into a discrete Fourier basis, that is, *e*^−*j*2*πmi*/*M*^ with *m* = 0, …, *M* − 1, *i* = 0, …, *M* − 1. The *M* matched filters can perform ordinary beamforming to obtain *M* orthogonal beams for omnidirectional detection. Since we focus on the feature of transmit beamspace, we adopt non-adaptive beamforming at the receiver. The output after receive beamforming can be written as:

(10) $$z(t,\theta )=w_{R}^{T}Y(t,\theta )$$

where ***w****_R_* = [1, 1, …, 1]*_N_*_×1_ is the receive weight vector, $\tau_{i}=2R_{i}/c,i=1,\ldots ,Q$. Then, the signal is fed back into the matched filter bank. The output of the *i*th matched filter channel can be written as:

(11) $$\tilde{z}(t,\theta_{i})=h(t,\theta_{i})⊗z(t,\theta )$$

where ⊗ denotes the convolution operator. Note that the matched filtering can also be conducted before receive beamforming. Two processing procedures are theoretically equivalent with differences in practical application. Here, we utilise receive beamforming before matched filter to reduce the system complexity. Next, substitute (8) and (10) into (11), the output is expanded as:

(12) $$\tilde{z}(t,\theta_{i})=\sum_{q=1}^{Q}\sum_{n=1}^{M}\sum_{m=1}^{M}\zeta_{q}e^{j2\pi \frac{d}{\lambda }(m-1)\sin\theta_{q}}e^{-j2\pi \frac{d}{\lambda }(n-1)\sin\theta_{i}}\cdot ACF_{S}(i_{q}-n+m)$$

where the auto-correlation function ACF can be extended as

(13) $$\begin{array}{cc} ACF_{S}(i_{q}-n+m) & =\exp(j\pi \mu (t-(m+n-2)\cdot \Delta t+\tau_{q})(t+(n-m)\cdot \Delta t-\tau_{q}))\\ & *\frac{\sin(2\pi \mu (T_{P}+(N-1)\cdot \Delta t)(t+(n-m)\cdot \Delta t-\tau_{q}))}{2\pi \mu (t+(n-m)\cdot \Delta t-\tau_{q})} \end{array}$$

The ACF is a function of both continuous time *τ_q_* and discrete time *i_q_*, *m*, *n* with *i_q_* = *τ_q_*/Δ*t*. The envelope in (13) is similar with *sinc* function. Besides, it is shown in (12) that the equivalent transmit beampattern via matched filtering is determined by the exponential term, and the time shift Δ*t* has no influence on it*.* Thereby, the angular resolution in CSTCA is the same as that in phased array counterpart.

**Figure 3:**
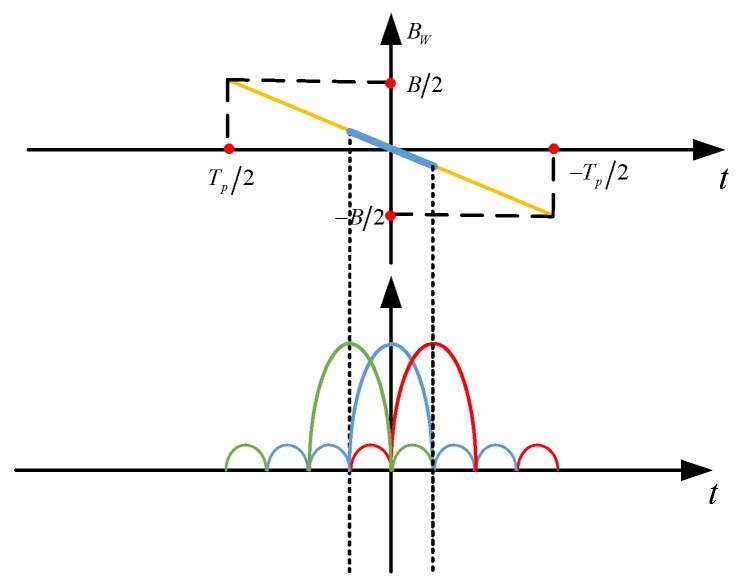
Sketch of equivalent transmit beampattern and the time-frequency relationship of LFM signal.

It is worth noticing that the space-time matched filter can bring an equivalent transmit beampattern gain with a value of 10log_10_*M*^2^*.* Additionally, the pulse compression gain is equal to 10log_10_*BT*/*M*, which is reduced by a factor of *M* in comparison with conventional pulse compression gain 10log_10_*BT.* The main reasons are listed as follows. At first, the power of transmitted signal is uniformly distributed in space. As a result, the power for one of the *M* beams is only 1/*M* of the total radiated power. Secondly, as shown in Figure 3, the time duration of the mainlobe (the segment between two dotted lines) is only the fraction 1/*M* of the whole pulse duration, since the width of mainlobe at a given probing angle is π/*M* in typical beampattern. Consequently, the effective coherent summation time of space-time matched filter is equal to *T_p_*/*M*, which means only 1/*M* of the bandwidth is sent in LFM signal. Thus, the pulse compression gain is degraded by a factor *M.* Furthermore, the range resolution is also reduced.

### 3.2. Beamspace MUSIC Algorithm

In this subsection, we propose a simple beamspace MUSIC algorithm. Unlike conventional beamspace DOA estimation methods, the spatial spectrum estimation in CSTCA can be directly performed in beamspace without design of beamforming matrix, since multiple beams are formed via space-time matched filtering.

Because different matched angles of filters give rise to different outputs. Peaks only appear via the matched filter wherein the matched angle is closest to the true target angle. For other matched filters, it is mismatched. We regard the peak locations as the target ranges. Thus, the target range can be considered as a priori knowledge. The samples at peaks are picked out. They serve as training samples to construct the sample covariance matrix (SCM). Firstly, we can obtain:

(14) $$z^{\prime }=\sum_{i=1}^{Q}\tilde{Z}(\tau_{i})$$

where $\tilde{Z}(t)={\left[\tilde{z}(t,\theta_{1}),\tilde{z}(t,\theta_{2}),\cdots ,\tilde{z}(t,\theta_{M})\right]}^{T}$, $\tau_{i}=2R_{i}/c,\;i=1,\ldots ,Q$. To acquire a sufficient amount of training samples, we exploit the coherent pulse train in slow time domain. Received data from *K* pulses is arranged into the *M*×*K* SCM, written as:

(15) $$Z^{\prime }=\left[z_{1}^{\prime },z_{2}^{\prime },\cdots ,z_{K}^{\prime }\right]$$

**Figure 4:**
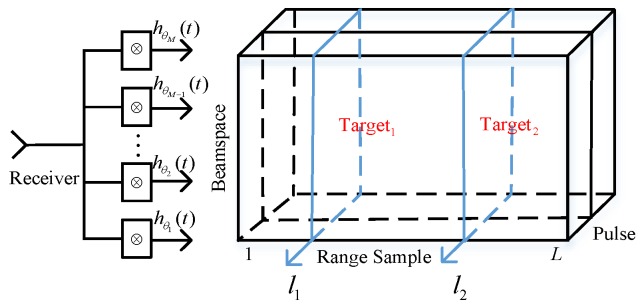
Matched filter configuration and data cube.

The data cube and matched filter configuration are portrayed in Figure 4 to visualize the processing. As can be seen, range cells of potential targets are selected and are combined with the *K* pulses. In practice, the SCM is basically estimated by secondary training samples referred to as sample matrix inverse (SMI) method, which is expressed as:

(16) $$\hat{R}=Z^{\prime }{Z^{\prime }}^{H}$$

where (·)^*H*^ denotes the Hermitian operation.

The eigen-decomposition of SCM $\hat{R}$ yields $\hat{R}=E\Lambda E^{H}=E_{S}\Lambda_{S}E_{S}^{H}+E_{N}\Lambda_{N}E_{N}^{H}$, where ***E**_S_* and ***E**_N_* are the signal subspace matrix and orthogonal noise subspace matrix, ***Λ**_S_* = *diag*{*λ*_1_, …, *λ_Q_*} and ***Λ**_N_* = *diag*{*σ*^2^, …, *σ*^2^} collect the eigenvalues with *λ*_1_ ≥ *λ*_2_ ≥ … ≥ *λ_Q_* ≥ *λ*_*Q*+1_ = … = *σ*^2^, respectively. Subsequently, the beamspace spatial spectrum is established as:

(17) $$P(\theta )=\frac{1}{a_{beam}^{H}(\theta )E_{N}{E_{N}}^{H}a_{beam}(\theta )}$$

where ***a****_beam_*(*θ*) is the *M ×* 1 beamspace steering vector. It is noteworthy that ***a****_beam_*(*θ*) is distinguishable with the element space steering vector ***a****_element_*(*θ*) = [1, exp{*j*2*πd*sin*θ/λ*},…, exp{*j*2*π*(*M* − 1)*d*sin*θ/λ*}]. We devise the beamspace steering vector by the following steps. Firstly, signal reflected from direction *θ*, range *R_q_* via *i*th matched filter can be written as:

(18) $$F_{i}\left(\theta ,t\right)=(\sum_{m=1}^{M}e^{j2\pi \frac{d}{\lambda }(m-1)\sin\theta }s(t-(m-1)\cdot \Delta t-\tau_{q})⊗(\sum_{n=1}^{M}e^{j2\pi \frac{d}{\lambda }(n-1)\sin\theta_{i}}s(t-(n-1)\cdot \Delta t))$$

Next, stack the outputs from *M* channels into a column vector:

(19) $$\tilde{F}\left(\theta ,t\right)={\left[F_{1}\left(\theta ,t\right),F_{2}\left(\theta ,t\right),\cdots ,F_{M}\left(\theta ,t\right)\right]}^{T}$$

Then, the samples in target range bins are selected out to make up the beamspace searching vector, that is:

(20) $$a_{beam}(\theta )=\tilde{F}\left(\theta ,\tau_{q}\right)$$

Since the pulse compression and beamforming are implemented concurrently, data via matched filtering is decoupled in time and angle domains. It implies that the range bin corresponding to any one of targets contains the angle information to reflect characteristics of the beamspace steering vector. Therefore, we only choose an arbitrary target to construct the beamspace searching vector in (20), which can significantly reduce the computational burden compared with utilization of entire *Q* targets. Moreover, the amplitudes of *Q* spectral peaks in (20) are different. The spectral peak intensity of the chosen *q*th target is slightly higher than others. When we use all the target samples to obtain beamspace steering vector, the peak intensities of *Q* targets will tend to be the same. However, it is insignificant to performance improvements and increases the computational complexity.

### 3.3. Spatial Smoothing Method in Transform Domain

In this subsection, we consider multiple closely spaced targets whose spacing is less than a range resolution cell. Since the range cell consisting of different target samples can’t satisfy the independent and identically distributed (IID) conditions and embodies feature of coherent signals, the proposed beamspace MUSIC method fails to estimate DOA accurately. To deal with this issue, we design a transformation matrix to apply spatial smoothing in element space.

Classical spatial smoothing technique makes use of the linear phase relationship between elements to guarantee nonsingularity of SCM. Motivated by the fact that the ordinary beamforming in beamspace via space-time matched filter is based on the discrete Fourier transform (DFT), we may conversely perform IDFT to return to element space. However, the beamforming matrix in space-time matched filter isn’t strictly in accordance with the discrete Fourier basis. Correspondingly, we can’t obtain the rotational invariance property in element space through IDFT directly. Remarkably, we design the transformation matrix with an approximate Vandermonde structure, which can be expressed as:

(21) $$T=\left[\begin{array}{cccc} 1 & 1 & \cdots & 1\\ e^{j2\pi \frac{d}{\lambda }\sin\theta_{1}} & e^{j2\pi \frac{d}{\lambda }\sin\theta_{2}} & \cdots & e^{j2\pi \frac{d}{\lambda }\sin\theta_{M}}\\ \vdots & \vdots & \vdots & \vdots \\ e^{j2\pi \frac{d}{\lambda }(M-1)\sin\theta_{1}} & e^{j2\pi \frac{d}{\lambda }(M-1)\sin\theta_{2}} & \cdots & e^{j2\pi \frac{d}{\lambda }(M-1)\sin\theta_{M}} \end{array}\right]$$

Thus, the data matrix in element space can be written as:

(22) $$X\left(t\right)=T\tilde{Z}\left(t\right)$$

**Figure 5:**
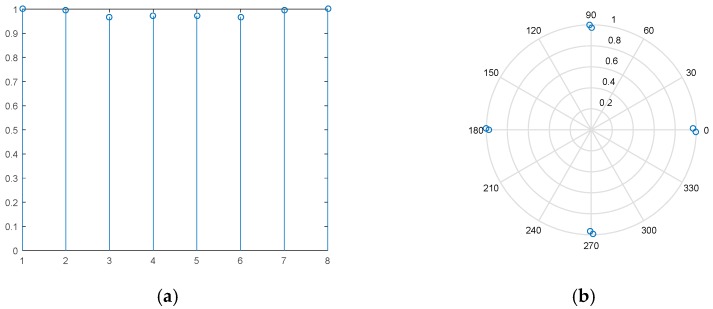
Illustration of linear relationship in element space with *M* = 8 (**a**) the amplitude diagram; (**b**) the phase diagram.

Figure 5 instantiates the regularity of amplitude-phase for data in element space. As can be seen, the amplitude is approximate to the unit value after transformation. Meanwhile, the phase is uniformly distributed at intervals of 90° in case of a single target at *θ*_0_ = 30°, *R*_0_ = 5 km. It verifies that the transformation procedure in (21) can restore the rotational invariance structure. In sequence, the range bins of targets are extracted out of data matrix ***X***. The *M* × 1 data vector can be given as:

(23) $$x^{\prime }=\sum_{i=1}^{Q}X\left(\tau_{i}\right)$$

where $\tau_{i}=2R_{i}/c,\;i=1,\ldots ,Q$. Then, we can obtain the *M × K* data matrix by sampling from *K* pulses, which is expressed as:

(24) $$X^{\prime }=\left[x_{1}^{\prime },x_{2}^{\prime },\cdots x_{K}^{\prime }\right]$$

Next, we divide the *M* elements uniform linear array (ULA) into *P* = *M* − *N*_0_ *+* 1 overlapping subarrays of size *N*_0_. Each subarray is separated by one element. The covariance matrix of the *p*th subarray can be written as:

(25) $${\hat{R}}_{p}=X_{p}^{\prime \prime }{X_{p}^{\prime \prime }}^{H}$$

where $X_{p}^{\prime \prime }={\left[X^{\prime }\left(p\right),X^{\prime }\left(p+1\right),\cdots ,X^{\prime }\left(p+N_{0}+1\right)\right]}^{T}$ is the *N*_0_ × *K* data matrix of the *p*th subarray, $X^{\prime }\left(p\right)$ denotes the *p*th row vector of $X^{\prime }$. The spatial smoothing matrix is the average of subarray covariance matrix, expressed as:

(26) $$\hat{R}=\frac{1}{P}\sum_{p=1}^{P}{\hat{R}}_{p}$$

Eventually, we can adopt basic MUSIC method to estimate DOAs since we have transformed data into the element space. Because of limitation of space, we won’t repeat it here.

## 4. Simulation Results

In this section, we consider a 13-element uniform linear CSTCA with half-wavelength spacing. The reference carrier frequency is *f*_0_ = 10 GHz and the time shift is Δ*t* = 0.05 µs. We set SNR = −20 dB unless otherwise specified. Note that the said SNR is before matched filtering. The additive noise is zero-mean complex Gaussian distributed. We use 0.001° mesh grids to search candidate peaks, and conduct computer simulations with 200 independent Monte Carlo trials. The accuracy of DOA estimates is evaluated by the root mean square errors (RMSEs), expressed as:

(27) $$\mathrm{RMSE}=\sqrt{\frac{1}{\mathrm{Mon}Q}\sum_{i=1}^{\mathrm{Mon}}\sum_{q=1}^{Q}{\left({\hat{\theta }}_{q}(i)-\theta_{q}\right)}^{2}}$$

where Mon denotes the number of Monte Carlo trial runs, ${\hat{\theta }}_{q}\left(i\right)$ is the estimated angle of the *q*th target in the *i*th run, and *θ_q_* is the true angle of the *q*th target. Another performance measurement metric is the resolution probability reflecting detection efficiency. In this case, we assume two closely-spaced point targets from directions *θ*_1_ = 4°and *θ*_2_ = 6°. It succeeds in resolving if and only if the following inequality holds [43]:

(28) $$\frac{D(\theta_{1})+D(\theta_{2})}{2}>D\left(\frac{\theta_{1}+\theta_{2}}{2}\right)$$

where $D(\theta_{i})=M-a_{beam}^{H}\left(\theta_{i}\right)E_{S}E_{S}^{H}a_{beam}\left(\theta_{i}\right)$ is the null spectrum value of *θ_i_*.

### 4.1. Two-Dimensional Space-Time Matched Filter

**Figure 6:**
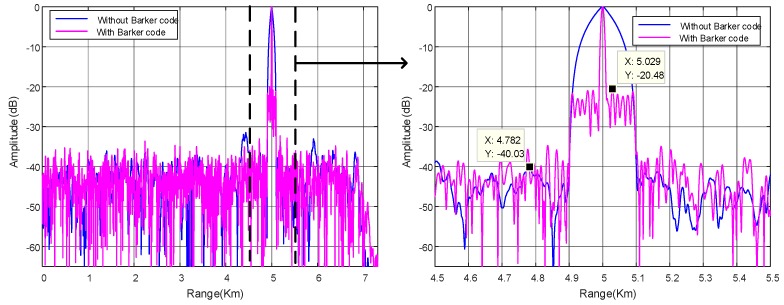
The one-dimensional range profile via space-time matched filtering for *θ*_1_ = 0° and *R*_1_ = 5 km.

Figure 6 plots the one-dimensional range profile via space-time matched filtering in case of a single target at *θ*_1_ = 0° and *R*_1_ = 5 km. The right side figure is the zoom-in of the range profile in range of [4.5 km, 5.5 km]. It is shown that the two peaks are situated at target locations. Note that the −3 dB width of the mainlobe for hybrid signal is evidently narrower than that for circulating LFM signal. As mentioned before in Section 3.1, the range resolution is degraded by a factor of *M* when transmitting circulating LFM signal in CSTCA. Particularly, the hybrid code scheme can significantly improve the range resolution. In addition, the sidelobe level for circulating LFM waveform is nearly below −40 dB in most range areas without window weighting. It is much lower than the SLL of LFM waveform itself, which is generally −13.2 dB. The reason is that transmitted signal of every element influences each other via sidelobes. As a result, the sidelobes can compensate each other for most ranges. Based on the positive impact of the sidelobes’ interaction, the SLL is reduced. However, the SLL for hybrid code is increased considerably, wherein it is almost 20 dB higher than that for circulating LFM signal. Since the time and space are dependent in CSTCA, both the spatial waveform and the temporal waveform are demanded to have low range SLLs to reduce the synthesized SLL. However, the peak SLL of spatial phase code is much higher than that for pure circulating temporal waveform, since the code length is limited by the element number. Thus, the peak SLL is larger for hybrid code. We can conclude that the range resolution enhancement in hybrid code is at cost of the side lobe level.

### 4.2. DOA Estimation Performance Analysis for Beamspace MUSIC Algorithm

In this example, we assess the performance of proposed beamspace MUSIC algorithm. The analytical expression for exact Cramer-Rao lower bounds (CRLBs) is derived to benchmark the method (see Appendix A).

**Figure 7:**
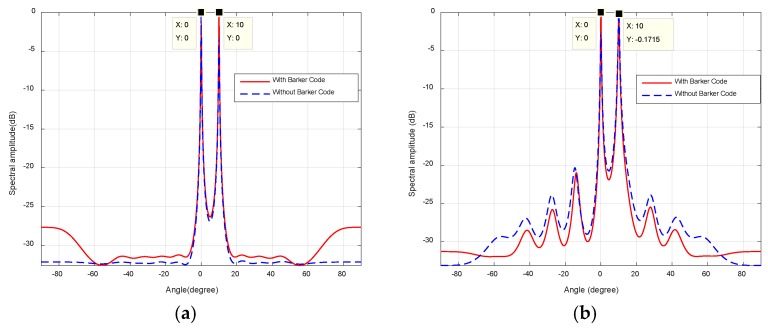
Comparison of the spatial spectral, (**a**) angles in (8) is uniformly distributed in form of sin(*θ*); (**b**) angles in (8) is uniformly distributed in range of [−π/2, π/2).

Assume that two targets are at *θ*_1_ = 0°, *θ*_2_ = 10°, and *R*_1_ = 3 km, *R*_2_ = 5 km. As shown in Figure 7, the proposed method can accurately estimate DOAs with two peaks at targets’ locations, that is, *θ*_1_ = 0°and *θ*_2_ = 10°. The beamforming angle in space-time matched filter is uniformly distributed in form of sin(*θ*) in Figure 7a in contrast of a uniform distribution of *θ* in Figure 7b*.* As can be seen, the performance in Figure 7a outperforms that in Figure 7b. Since the steering vector $e^{j2\pi \left(n-1\right)d\sin\theta i/\lambda }$ in matched filter is consistent with Discrete Fourier orthogonal basis in Figure 7a. As a result, the transformation from element space to beamspace via matched filtering is an orthogonal transformation, which can ascertain no loss of energy in the process. Therefore, the corresponding sample covariance matrix can provide better orthogonality between noise subspace and beamspace steering vector in (17). Moreover, there is little discrepancy between the two paradigms. Because the imposed Barker code alters the original phases of circulating LFM signal without any changes in the waveform itself.

**Figure 8:**
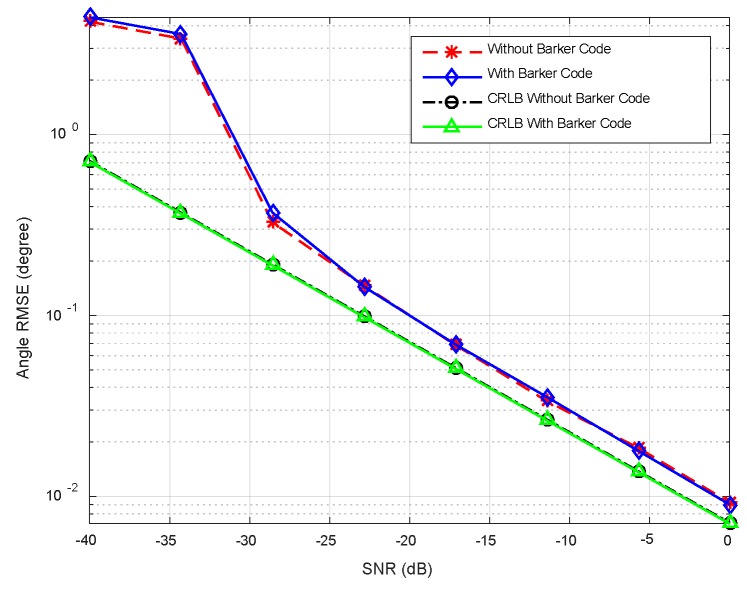
Comparison between CRLB and RMSE versus SNRs.

**Figure 9:**
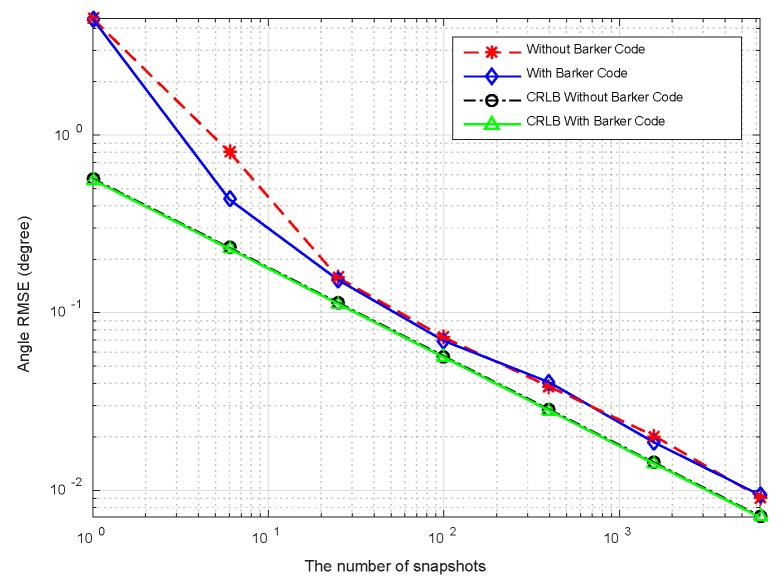
Comparison between CRLB and RMSE versus number of snapshots.

In this example, we consider two targets at *θ*_1_ = −3°, *θ*_2_ = 5°, respectively. The sample number *K* is set to be 100. The pulse compression gain is approximate to 10 dB, and the equivalent transmit beampatten gain is nearly 20 dB. Therefore, overall gain is 30 dB. The RMSEs with respect to input SNRs are plotted in Figure 8, where the SNRs vary over a range of −40 dB to 0 dB. It can be observed that the RMSEs decrease monotonically as SNRs increase. A little difference exists between the two cases (with or without Barker code). It is seen from Figure 8 that RMSEs of DOA estimation are close to the CRLBs at moderate and high SNRs regions. The accuracy of the angle estimation is enhanced with increment of SNRs. Figure 9 plots RMSEs versus the number of snapshots at SNR = −20 dB. It is shown that the angle can be estimated accurately at small number of snapshots. The direction finding performance attains the CRLBs when the snapshot number is more than 20.

**Figure 10:**
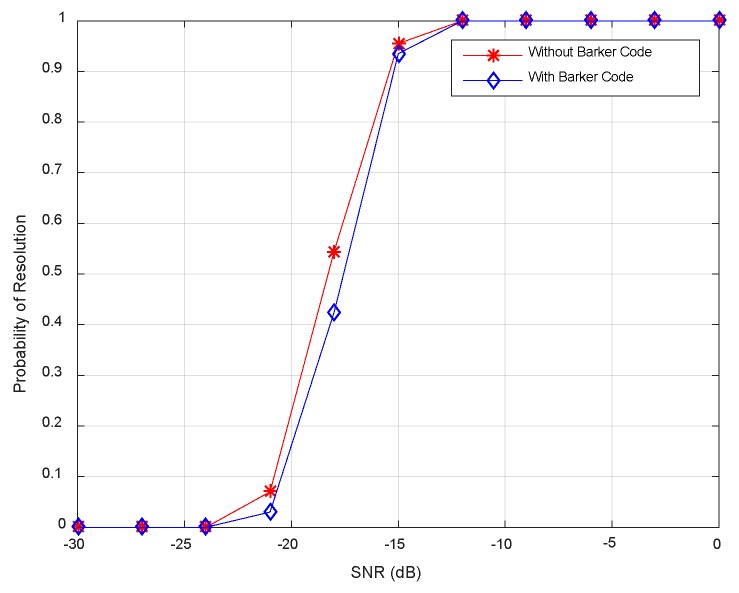
Resolution probability against SNRs.

**Figure 11:**
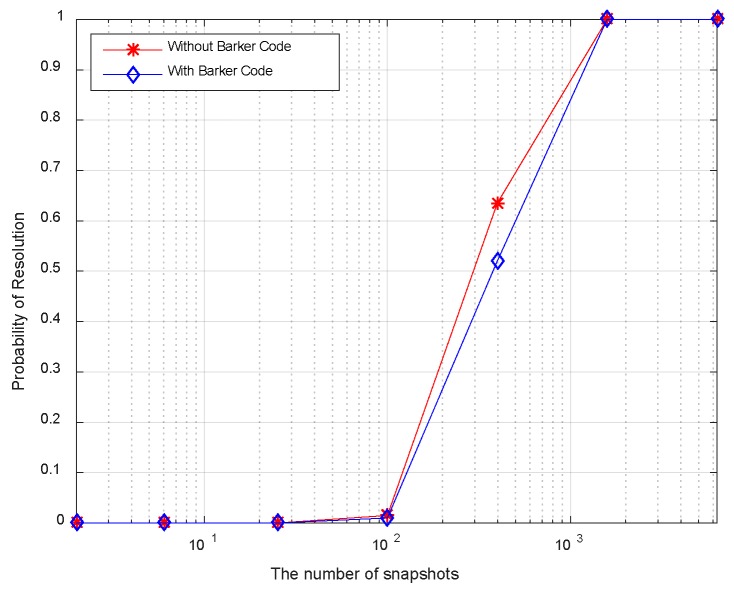
Resolution probability against number of snapshots.

**Figure 12:**
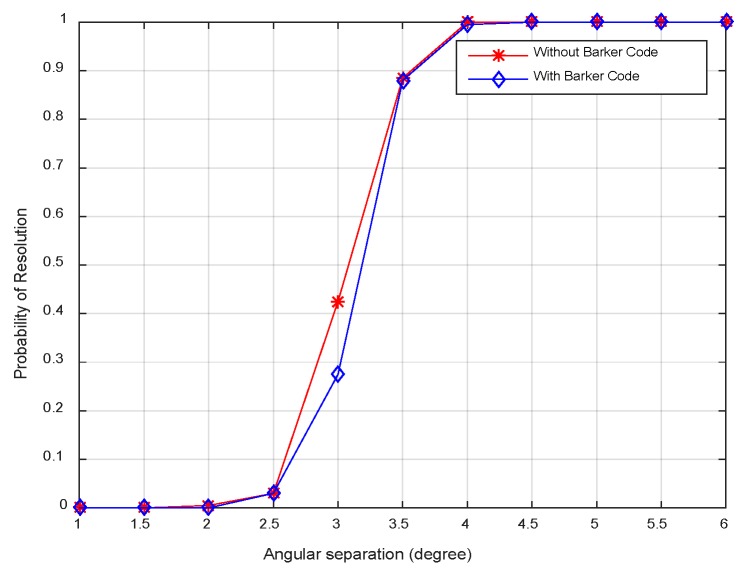
Resolution probability against angular separations.

Figure 10 displays the resolution probability against SNRs at *K* = 50. It is shown that the resolution probability becomes larger when SNRs increase. It achieves 100% at SNR = −18 dB. In Figure 11, the variation of resolution probability with number of snapshots is depicted at SNR = −20 dB. It is observed that the resolution probability increases with a larger sample number. In Figure 12, the dependence between resolution probability and the angle separation is plotted at SNR = −20 dB and *K* = 50. The angles of two targets are set as *θ*_1_ = *θ*_cen_ − Δ*θ*/2 and *θ*_2_ = *θ*_cen_ + Δ*θ*/2 with central angle *θ*_cen_ = 5°. The angle separation is from 1° to 6°. The minimum angular separation for a specific resolution performance can be determined from the curves. It is shown that the resolution probability increases monotonically with increased angle separations in the range of [2.5°, 4°]. In addition, measurements of two transmit waveforms show slightly differences in DOA estimation performance in Figure 10, Figure 11 and Figure 12.

### 4.3. DOA Estimation Performance Analysis for Spatial Smoothing in Transform Domain

In this example, we demonstrate the effectiveness of the spatial smoothing technique in transform domain in case of two targets in the same range cell under test (CUT). The CSTCA is divided into 3 overlapped subarrays. The other parameters are identical with those in beamspace MUSIC simulations.

**Figure 13:**
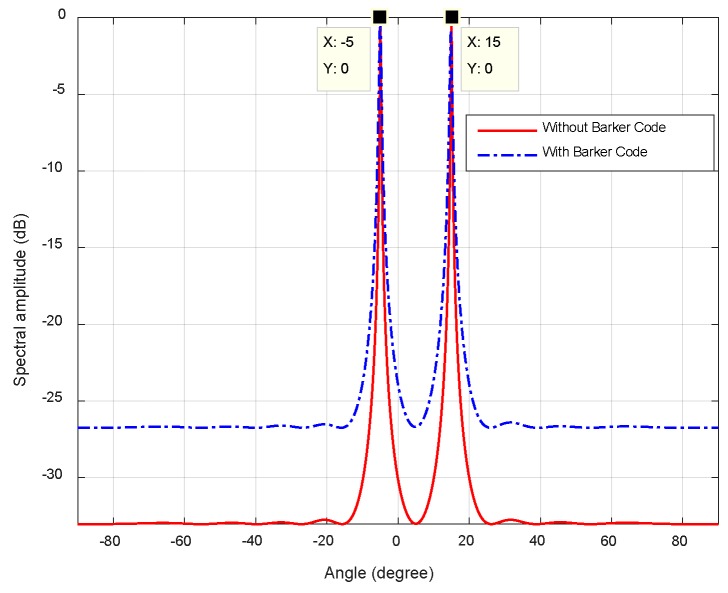
DOA estimation spatial spectrum using spatial smoothing technique in transform domain.

Figure 13 plots the spatial spectrum of two transmit waveforms. It is observed that the spectral peaks point to true target angles at *θ*_1_ = −5°, *θ*_2_ = 15° and *R*_1_ *= R*_2_ = 5 km, respectively. Moreover, the spatial spectrum of pure circulating LFM waveform has lower background noise level and more evident peak than the hybrid code. The reason is that Barker code introduces random phases across array elements, which has adverse influence on spatial phase relationship in element domain. In this regard, pure circulating LFM waveform is more beneficial to angle estimation.

**Figure 14:**
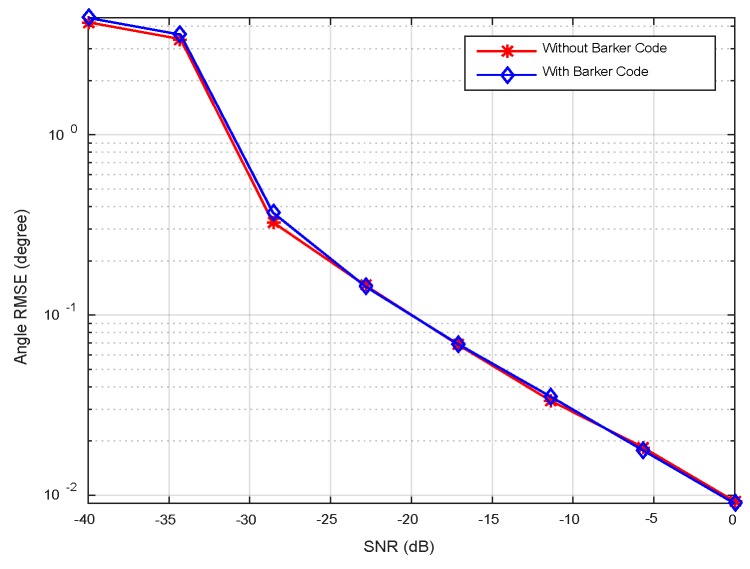
RMSEs versus the SNRs.

**Figure 15:**
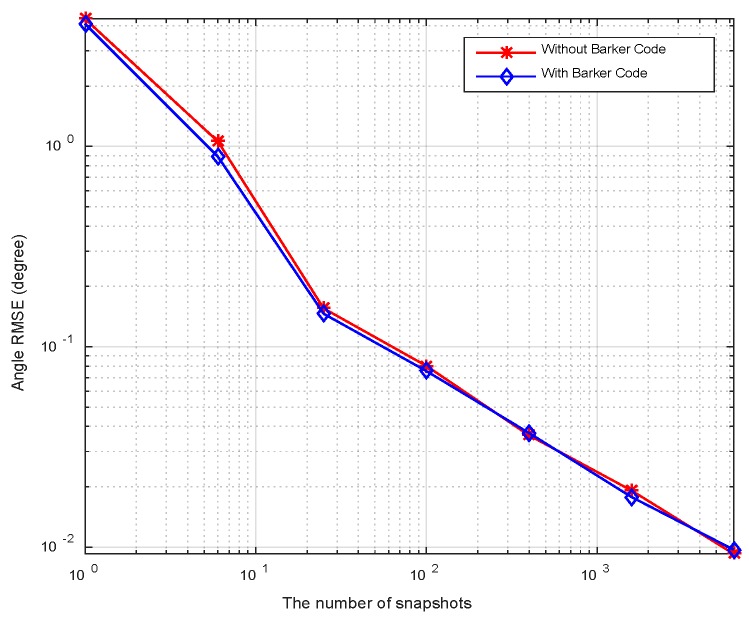
RMSEs versus the number of snapshots.

The RMSEs curves versus SNRs at *K* = 50 are displayed in Figure 14. It is shown that the angular RMSEs descend linearly when SNR > −30 dB demonstrating the excellent performance in angular estimation. Figure 15 plots RMSEs curves versus snapshots, wherein two curves exhibit similar variation trends in comparison with Figure 9. Furthermore, the CRLB curve in element space is analogous to that in beamspace in Figure 8 and Figure 9 for the following two reasons. Firstly, the transformation from beamspace to element space through (21) doesn’t introduce additional information such as new independent training samples. Thereby, the structure of SCM isn’t changed. Secondly, the total energy is lossless in the orthogonal transformation step by (21). The reduced array aperture in subarray partition would cause a little gain loss. For the sake of simplicity, the CRLB curve in element space isn’t derived here.

**Figure 16:**
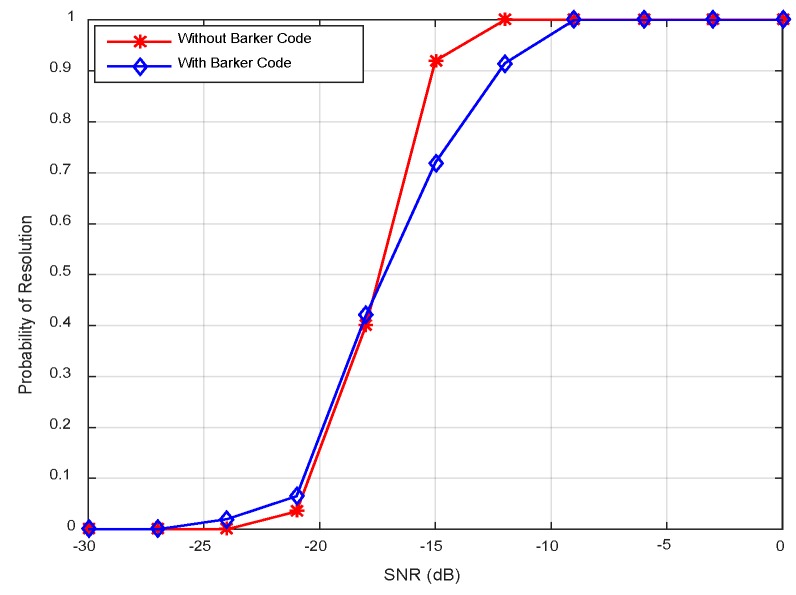
Resolution probability against SNRs.

**Figure 17:**
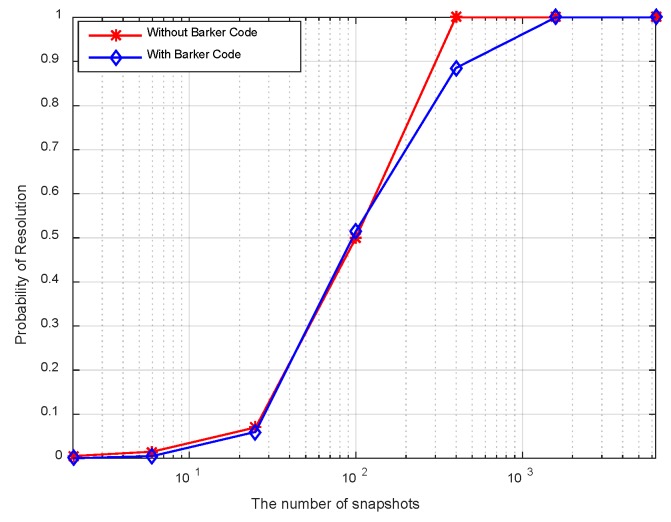
Resolution probability against the number of snapshots.

**Figure 18:**
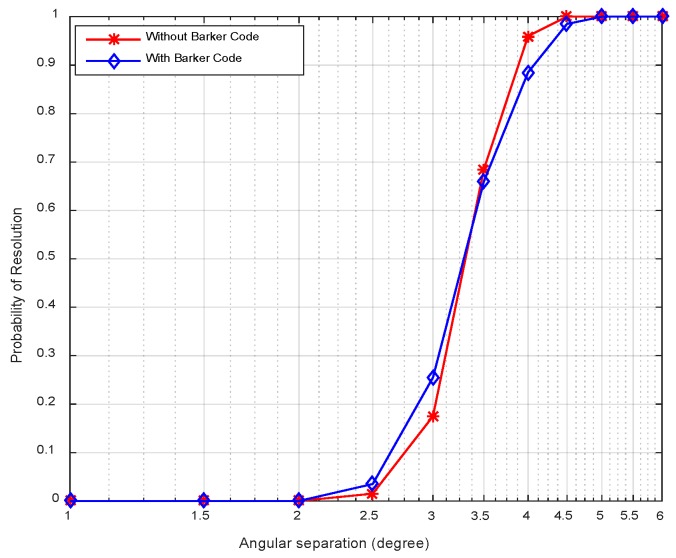
Resolution probability against the angular separation.

The resolution probability curves versus input SNRs, the number of snapshots and the angular separation are depicted in Figure 16, Figure 17 and Figure 18, respectively. As can be seen in Figure 16, the resolution probability for pure circulating space-time LFM signal is equal to 100% when SNRs ≥ −12 dB with *K* = 200. While it is −9 dB for the Barker code. It indicates that the circulating space-time LFM signal is more sensitive to the variation of SNRs. As illustrated in Figure 17, the probability of resolution is improved as the increase of snapshot number with SNRs = −25 dB. It is worth noting that the coherent pulse trains as sample data can also be applied to Doppler processing subsequently in future work. As shown in Figure 18, a good performance can be achieved when the angular separation is in excess of 4° in both curves.

## 5. Conclusions

The circulating space-time coding array (CSTCA) radar employs a tiny time shift across array elements. It can provide a simple way to acquire a wide angular coverage with a stable gain by transmitting an identical waveform, and obtains a low sidelobe level in range domain. As a special type of coherent colocated MIMO radar, CSTCA is much simpler to be implemented in practice. In this paper, we solve the issue of DOA estimation in CSTCA. At first, we designed a two-dimensional space-time matched filter to form multi-beams affording controllable transmit freedom, and the pulse compression is performed simultaneously. Then, we proposed the beamspace MUSIC method to estimate DOAs. We devised the beamspace searching vector to derive the spatial spectrum. Afterwards, we designed a transformation matrix to map the received data cube from beamspace into element space in case of closely-spaced targets. Based on the transformation, the RIP can be restored and spatial smoothing is performed. Theoretical performance analysis and numerical simulations on the RMSEs, the probability of resolution as well as CRLB curve can demonstrate the effectiveness of proposed approaches. Our future work will focus on the practical obstacles including DOA estimation under additive colored Gaussian noise scenarios. Multiple parameters optimization problem for performance enhancement is also one of ongoing investigations.

## Appendix A. CRLB Derivation for Angle Estimation

In this section, we study the performance lower bound of the CSTCA radar by deriving the CRLBs for angle. The unknown parameter vector can be modeled as $\alpha ={\left[\theta ,\bar{\zeta },\tilde{\zeta }\right]}^{T}$, where the deterministic parameters of the signal are collected in α with ζ¯=Re{ζ} and ζ˜=Im{ζ}, respectively. The likelihood function of the data in beamspace after matched filtering is given by:

(A1) $$L(\tilde{z}(1),\cdots ,\tilde{z}(K))=\frac{1}{{\left(2\pi \right)}^{K}{\left(\sigma /2\right)}^{K}}\exp\left\{-\frac{1}{\sigma }\sum_{k=1}^{K}{\left(\tilde{z}(k)-\tilde{y}(k)\right)}^{H}\cdot (\tilde{z}(k)-\tilde{y}(k))\right\}$$

where $\tilde{z}\left(k\right)$ denotes the *k*th column of $Z^{\prime }$ in (15), $\tilde{y}\left(k\right)$ denotes the signal components of $Z^{\prime }$. Then, the log-likelihood function is:

(A2) $$\ln L=-K\ln(\pi \sigma )-\frac{1}{\sigma }\sum_{k=1}^{K}{\left[\tilde{z}(k)-\tilde{y}(k\right]}^{H}\cdot \left[\tilde{z}(k)-\tilde{y}(k\right]$$

First, we calculate the derivatives of (30) with respect to θ, $\bar{\xi }$ and $\tilde{\xi }$. We have:

(A3) ∂lnL∂θ=2σRe{∑k=1K(∑m=1M∑n=1Mζ∂A∂θG)He}

(A4) ∂lnL∂ζ¯=2σRe{∑k=1K(∑m=1M∑n=1MAG)He}

(A5) ∂lnL∂ζ˜=2σIm{∑k=1K(∑m=1M∑n=1MAG)He}

where $A={\left[e^{j2\pi \frac{d}{\lambda }\left((m-1)\sin\theta -(n-1)\sin\theta_{1}\right)},\cdots ,e^{j2\pi \frac{d}{\lambda }\left((m-1)\sin\theta -(n-1)\sin\theta_{M}\right)}\right]}^{T}$ with $\sin\theta_{i}=-1+2(i-1)/M$, i=1,⋯,M and $G=e^{\left(-j\pi \mu (m+n-2)\cdot (n-m)\cdot \Delta t^{2}\right)}*\frac{\sin(\pi \mu T_{P}(n-m)\cdot \Delta t)}{\pi \mu (n-m)\cdot \Delta t}$, $e=\tilde{z}(k)-\tilde{y}(k)$ denotes the independent noise which is assumed to be a zero-mean white Gaussian vector with complex covariance $e=\sigma^{2}I_{M\times 1}$. In subsequence, the elements of the Fisher matrix ***J*** can be calculated by:

(A6) Jθθ=E[(∂lnL∂θ)(∂lnL∂θ)T]=2σRe{∑k=1K[(∑m=1M∑n=1Mζ∂A∂θG)H⋅(∑m=1M∑n=1Mζ∂A∂θG)]}

(A7) Jθζ¯=E[(∂lnL∂θ)(∂lnL∂ζ¯)T]=2σRe{∑k=1K[(∑m=1M∑n=1Mζ∂A∂θG)H⋅(∑m=1M∑n=1MAG)]}

(A8) Jθζ˜=E[(∂lnL∂θ)(∂lnL∂ζ˜)T]=−2σIm{∑k=1K[(∑m=1M∑n=1Mζ∂A∂θG)H⋅(∑m=1M∑n=1MAG)]}

(A9) Jζ¯ζ¯=E[(∂lnL∂ζ¯)(∂lnL∂ζ¯)T]=2σRe{∑k=1K[(∑m=1M∑n=1MAG)H⋅(∑m=1M∑n=1MAG)]}

(A10) Jζ¯ζ˜=E[(∂lnL∂ζ¯)(∂lnL∂ζ˜)T]=−2σIm{∑k=1K[(∑m=1M∑n=1MAG)H⋅(∑m=1M∑n=1MAG)]}

(A11) Jζ˜ζ˜=E[(∂lnL∂ζ˜)(∂lnL∂ζ˜)T]=2σRe{∑k=1K[(∑m=1M∑n=1MAG)H⋅(∑m=1M∑n=1MAG)]}

Define two vectors $w=\left(\sum_{m=1}^{M}\sum_{n=1}^{M}AG\right)$ and $w_{\theta }=\left(\sum_{m=1}^{M}\sum_{n=1}^{M}\frac{\partial A}{\partial \theta }G\right)$. Therefore, the FIM can be derived as:

(A12) J=[JθθJθζ¯Jθζ˜Jθζ¯HJζ¯ζ¯Jζ¯ζ˜Jθζ˜HJζ¯ζ˜HJζ˜ζ˜]=2Kσ[|ξ|2‖wθ‖2Re{wθHwζ∗}−Im{wθHwζ∗}Re{wθHwζ∗}‖w‖20−Im{wθHwζ∗}0‖w‖2]

By utilizing the Schur complement, the inverse of the FIM can be expressed as:

(A13) $$J^{-1}={\left[\begin{array}{cc} J_{11} & J_{12}\\ J_{21} & J_{22} \end{array}\right]}^{-1}=\left[\begin{array}{cc} D^{-1} & \times \\ \times & \times \end{array}\right]$$

where $J_{11}=J_{\theta \theta }$, $J_{12}=J_{21}^{H}=\left[\begin{array}{cc} J_{\theta \bar{\zeta }} & J_{\theta \tilde{\zeta }} \end{array}\right]$, $J_{22}=\left[\begin{array}{cc} J_{\bar{\zeta }\bar{\zeta }} & J_{\bar{\zeta }\tilde{\zeta }}\\ J_{\bar{\zeta }\tilde{\zeta }}^{H} & J_{\tilde{\zeta }\tilde{\zeta }} \end{array}\right]$. Thus, we can obtain:

(A14) $$D=J_{11}-J_{12}J_{22}^{-1}J_{21}=\frac{2K}{\sigma }{\left|\zeta \right|}^{2}\left({\left\|w_{\theta }\right\|}^{2}-\frac{{\left|w_{\theta }^{H}w\right|}^{2}}{{\left\|w\right\|}^{2}}\right)$$

The CRLBs for angle can be written as:

(A45) $$CRLB_{\theta }=\frac{1}{2K\cdot SNR\left({\left\|w_{\theta }\right\|}^{2}-\frac{{\left|w_{\theta }^{H}w\right|}^{2}}{{\left\|w\right\|}^{2}}\right)}$$
